# Does good leadership buffer effects of high emotional demands at work on risk of antidepressant treatment? A prospective study from two Nordic countries

**DOI:** 10.1007/s00127-014-0836-x

**Published:** 2014-02-20

**Authors:** Ida E. H. Madsen, Linda L. Magnusson Hanson, Reiner Rugulies, Töres Theorell, Hermann Burr, Finn Diderichsen, Hugo Westerlund

**Affiliations:** 1 The National Research Centre for the Working Environment, Lerso Park Allé 105, 2100 Copenhagen, Denmark; 2 Stress Research Institute, Stockholm University, Stockholm, Sweden; 3 Department of Public Health, University of Copenhagen, Copenhagen, Denmark; 4 Department of Psychology, University of Copenhagen, Copenhagen, Denmark; 5 Federal Institute of Occupational Safety and Health, Berlin, Germany

**Keywords:** Work, Stress, Mental health, Common mental disorder, Depression

## Abstract

**Purpose:**

Emotionally demanding work has been associated with increased risk of common mental disorders. Because emotional demands may not be preventable in certain occupations, the identification of workplace factors that can modify this association is vital. This article examines whether effects of emotional demands on antidepressant treatment, as an indicator of common mental disorders, are buffered by good leadership.

**Methods:**

We used data from two nationally representative work environment studies, the Danish Work Environment Cohort Study (*n* = 6,096) and the Swedish Longitudinal Occupational Survey of Health (*n* = 3,411), which were merged with national registers on antidepressant purchases. All individuals with poor self-reported baseline mental health or antidepressant purchases within 8.7 months before baseline were excluded, and data analysed prospectively. Using Cox regression, we examined hazard ratios (HRs) for antidepressants in relation to the joint effects of emotional demands and leadership quality. Buffering was assessed with Rothman’s synergy index. Cohort-specific risk estimates were pooled by random effects meta-analysis.

**Results:**

High emotional demands at work were associated with antidepressant treatment whether quality of leadership was poor (HR = 1.84, 95 % CI 1.32–2.57) or good (HR = 1.70, 95 % CI 1.25–2.31). The synergy index was 0.66 (95 % CI 0.34–1.28).

**Conclusions:**

Our findings suggest that good leadership does not substantially ameliorate any effects of emotional demands at work on employee mental health. Further research is needed to identify possible preventive measures for this work environment exposure.

## Introduction

Common mental disorders such as depression and anxiety are highly prevalent, and a leading cause of disease burden [1]. The causes of common mental disorder are thought to involve a complex interplay of social, psychological, and biological factors [2], and numerous studies have shown associations between psychosocial work environment factors and common mental disorder, particularly depression [3–8].

Emotional demands at work, i.e. aspects of the job that require sustained emotional effort [9], have been associated poor mental health outcomes, including psychological distress [10, 11] and depressive symptoms [12, 13]. Studies have also shown associations between emotional demands and indicators of clinically significant common mental disorders, such as hospitalisation with depression [14] and antidepressant treatment [15, 16]. Employees in human service work, such as health care, education, and social work have high emotional demands [15]. In these jobs, emotional demands are thought to stem, among other factors, from particular characteristics of the work, such as confrontation with the problems and suffering of patients and clients [17]––the needs of whom is the central focus of the work [18]. Hence, some types of work might be inherently emotionally demanding, and emotional demands may in these jobs not be amenable to change. It is, therefore, pivotal to examine whether the effects of emotional demands on ill-health can be buffered by other factors, which are modifiable.

The conservation of resources theory states that negative emotional outcomes may occur when resources that an individual values are threatened or lost [19]. Emotional demands at work may result in prolonged expenditure of emotional resources or energy, as they are aspects of the job that require sustained emotional effort [9]. According to conservation of resources theory, the availability of resources determines the impact of resource loss [19]. The theory further states that acknowledgement of accomplishments and tasks and understanding from superiors are important resources in a Western context [19]. Hence, leadership quality may be an important work environment resource, which could potentially buffer detrimental effects of emotional demands on employee mental health. Such buffering would be in line with previous research showing that positive leader behaviours, such as support, feedback, and trust, are associated with better employee well-being and help employees cope with stress [20, 21]. The potential of leaders to aid employee coping may also be inferred from the social support literature. If supportive leadership is considered a type of social support, then previous literature indicates that such behaviour may ameliorate the impact of stressful experiences [22, 23]. According to Thoits [22], buffering by social support may occur because supportive others give active coping assistance or provide emotional sustenance. In a work context, the leader likely has previous experience with the demands faced by the employee and could be considered an experientially similar other. Such individuals may offer emotional sustenance in terms of empathic understanding, acceptance of ventilation, and validation of feelings and concerns [22]. They may also be particularly suited for active coping assistance such as threat re-appraisal or offering of information and advice [22]. Finally, they may be a source of social comparison, offering a role model in dealing with the situation [22].

This study combines questionnaire and register data from two representative cohorts from Denmark and Sweden, respectively. The aim of the study was to examine whether the association between emotional demands and common mental disorders can be modified by good leadership. Specifically, we hypothesise that the effect of emotional demands on incident antidepressant treatment––as an indicator of common mental disorder––is buffered by good leadership quality, i.e. that the joint effect of these two factors is less than additive. We further explore whether the associations are similar in these two Scandinavian countries.

## Methods

### Study design

This study uses data from two representative Scandinavian cohort studies, the Danish Work Environment Cohort Study (DWECS), and the Swedish Longitudinal Occupational Survey of Health (SLOSH). From these multi-wave studies we used DWECS 2005 and SLOSH 2006 because they were collected at a similar time. The questionnaire data were linked with national registers on medication purchases [24]. This study was conducted in accordance with the Helsinki declaration and all individuals gave informed consent to participate in the respective studies by responding to the interview or questionnaire. SLOSH has been approved by the regional ethics board. DWECS has been approved by the Danish Data Protection Agency, but approval from the Danish National Committee on Biomedical Research Ethics is not required for Danish questionnaire- and register-based studies [25].

Details of the included cohort studies are published elsewhere [26–28]. Briefly, DWECS is an on-going cohort study with 5-year follow-ups since 1990. At each wave, the cohort is supplemented to ensure representativeness of the Danish working population. Data for DWECS 2005 were collected during October 2005–May 2006. There were 19,855 eligible participants, of whom 12,413 (62.5 %) responded. Of these individuals, 8,064 individuals were gainfully employed, aged 20–59 years, and had full information on personal identification number. The age range was chosen for similarity to SLOSH, which included only individuals 20 years or older, and to include individuals within the average working age (<60 years).

SLOSH is an on-going cohort study that started as a follow-up of individuals from the Swedish Work Environment Survey (SWES) from 2003 to 2007. Respondents to SWES, largely representative of Swedish citizens in gainful employment aged 16–64, were invited to participate in SLOSH. Data for SLOSH were collected during March–May 2006 by inviting the 9,154 eligible participants in SWES 2003, of which 5,985 (65 %) responded. Of these individuals, 4,351 were gainfully employed (30 % or more on average during the past 3 months) and aged 20–59 years.

### Measuring antidepressant treatment

Antidepressant treatment was measured through national registers on prescription medication purchases, namely the Danish register of medicinal product statistics and the Swedish prescribed drug register [24]. These registers contain all purchases of prescription medications at pharmacies in Denmark since 1995 and Sweden since July 2005. For the present analyses we included Danish data from January 2005 to December 2008 and Swedish data from July 2005 to April 2009. To ensure a prospective analysis on incident episodes of antidepressant treatment, we excluded individuals with any purchase of antidepressants 8.7 months (263 days) before baseline. Time of follow-up was 2.6 years (960 days). The periods for follow-up and exclusion of prevalent cases were chosen because they were the maximum available for both cohorts.

### Measuring emotional demands and leadership quality

The items used to measure emotional demands at work and leadership quality are presented in Table 1. Emotional demands were assessed in both studies using an item from the Copenhagen Psychosocial Questionnaire (COPSOQ) [29]. To assess leadership quality we constructed a four-item scale measuring whether the manager listens is supportive, appreciative, and informative. The scales’ dimensions and items were chosen partly for commonality between the two cohorts.

**Table 1:** Items measuring emotional demands and leadership quality

Construct	Denmark				Sweden	
	Item	Response options	Source	Item	Response options	Source
Emotional demands	Does your work put you in emotionally disturbing situations?	Always Often Sometimes Seldom Never/hardly ever	COPSOQ, emotional demands	Does your work put you in emotionally disturbing situations?	Often Sometimes Seldom Never/hardly ever	COPSOQ, emotional demands
Leadership quality						
Listens	How often is your immediate superior willing to listen to your work-related problems?	Always Often Sometimes Seldom Never/hardly ever	COPSOQ, social support from supervisor	Does your manager listen to you and pay attention to what you say?	To a very small extent/not at all To a small extent To a high extent To a very high extent	NA [MW], relations with immediate manager
Supportive	How often do you get help and support from your immediate superior?	Always Often Sometimes Seldom Never/hardly ever	COPSOQ, social support from supervisor	Can you receive support and encouragement from your superiors when your work becomes troublesome?	Never Mostly not Mostly Always	SWES
Appreciative	Is your work acknowledged and appreciated by the management?	To a very high degree To a high degree neither/nor To a low degree To a very low degree	DWECS, rewards	Do you receive affirmation from your manager?	Hardly ever/never No, seldom Yes, sometimes Yes, often	NA [MW], relations with immediate manager
Informative	At your place of work, are you informed well in advance concerning for example important decisions, changes, or plans for the future?	To a very large extent To a large extent Somewhat To a small extent To a very small extent	COPSOQ, predictability	At my work place we are informed well in advance of important decisions	Strongly disagree Somewhat disagree Strongly agree	NA [MW], workplace democracy

*COPSOQ* Copenhagen Psychosocial Questionnaire [54], *DWECS* Danish Work Environment Cohort Study [26], *NA* Nya Arbetslivet (Modern Worklife) [55], *SWES* Swedish Work Environment Study [56]

We dichotomized emotional demands by defining the responses Sometimes, Often, and Always as high emotional demands. This categorization was based on the distribution of the respondents in the two samples, as the chosen cut off point resulted in about half of the respondents being defined as having high emotional demands. As the samples were nationally representative, we considered an approximate median split a meaningful way of distinguishing high and low levels of emotional demands. Based on the leadership items a composite mean score ranging from 0 through 100 was constructed. This scale was dichotomised at the cohort specific median to define good versus poor quality of leadership.

### Covariates

We included sex and age from the Danish Civil Registration system [30] for DWECS and from self-report for SLOSH. Income was obtained from Statistics Denmark and Sweden, and deaths from the Danish and Swedish Registers of causes of deaths. We assessed education by register data in DWECS [31] and by self-report in SLOSH, and categorised the variable into ≤9, 10–11, 12, 13–14, and ≥15 years of schooling. We further included self-reported data on marital status (living with a partner or spouse, yes/no), employment status (part-time/full-time), and mental health at baseline. Part-time work was defined as working <37 h in DWECS and <37 or 40 h in SLOSH, depending on occupation.

In DWECS, mental health at baseline was assessed by the five-item Mental Health Inventory (MHI-5) from the Short Form 36 questionnaire [32]. In SLOSH, mental health was assessed by six questions from the (Hopkins) Symptom Checklist 90 (SCL-90) [33]. Scales for mental health were derived as the mean value of the items. Individuals with poor mental health at baseline, defined as MHI-5 ≤52 (scored from 0 to 100) or SCL ≥3.75 (scored from 1 to 5, corresponding to a summary score of ≥17 on a scale from 0 to 24 and about 5 % of the populations), were excluded from the analyses to limit reverse causality. Such reverse causality might occur if individuals with pre-existing poor mental health at baseline were also more likely to experience their work as emotionally demanding.

For the sensitivity analyses we further included occupation according to the major groupings of the International Standard Classification of Occupations from 1988 [34]: (0) armed forces, (1) legislators, senior officials, and managers, (2) professionals, (3) clerks, (4) service workers and shop and market sales workers, (5) skilled agricultural and fishery workers, (6) craft and related trades workers, (7) plant and machine operators and assemblers, and (8) elementary occupations.

## Study population

### DWECS 2005

There were 8,064 gainfully employed DWECS participants aged 20–59 years. We excluded 696 individuals who were self-employed, as quality of leadership was not relevant for this group. Further, we excluded 319 individuals with previous purchases of antidepressants, 336 individuals with poor self-reported mental health at baseline, and 623 individuals with missing data on covariates. The final study sample included 6,096 DWECS participants.

### SLOSH 2006

There were 4,318 gainfully employed SLOSH participants aged 20–59 years. After excluding 244 self-employed individuals, we excluded 248 individuals with previous purchases of antidepressants, 183 with poor self-reported mental health at baseline, and 232 with missing data on covariates. The final study sample included 3,411 SLOSH participants.

### Statistical analyses

Using Cox proportional hazards regression we examined relative hazard rates [i.e. hazard ratios (HR)] of entering antidepressant treatment during 2.6 years (960 days) of follow-up. Participants were followed in the registers until first purchase of antidepressants, death, or end of follow-up, whichever came first. Analyses controlled for sex, age, marital status, education, income, and employment status. These potential confounders were chosen a priori as they have been associated with common mental disorder [35–37] and could be associated with the examined exposures.

The proportional hazards assumption was tested visually by inspecting the cohort-specific log–log survival plots. Following Rothman [38], we examined joint effects of emotional demands and good leadership by categorising respondents in four groups according to their exposure to these two factors. Both emotional demands and leadership quality were dichotomised and the group with low emotional demands and poor leadership used as reference. We examined interaction as departure from additivity using Rothman’s synergy index [38]. A synergy index smaller than one indicates that the joint effect is less than additive (antagonistic), suggesting that good leadership buffers the effect of emotional demands on antidepressant treatment. The 95 % confidence interval for the synergy index was constructed according to Hosmer and Lemeshow [39].

Country-specific risk estimates and standard errors were calculated using SAS version 9.2 (SAS Institute, Cary, NC, USA). Estimates were pooled using inverse variance weighted random effects meta analysis in R (version 2.10, http://www.rproject.org), applying the meta package [40]. The random effects estimate was based on the DerSimonian and Laird estimator [41], and heterogeneity between the country-specific risks estimates was assessed by Cochran’s *Q* test. In case of statistically significant heterogeneity (*p* < 0.10) the degree of heterogeneity was assessed by *I* ^2^ [42].

### Sensitivity analyses

To assess the robustness of the findings we conducted five sensitivity analyses: (1) examining whether findings were similar for men and women; (2) adjusting the main effects of emotional demands and leadership quality on antidepressants for baseline mental health. This adjustment assessed confounding of the associations by negative affect at baseline and potential differential misclassification of the self-reported work environment; (3) adjusting for occupation to assess confounding by occupation-related treatment-seeking behaviours; (4) examining the country-specific risk estimates for the joint effects of emotional demands and quality of leadership on antidepressants. This analysis assessed more qualitatively any differences between the countries; and (5) examining the influence of the cut-off point for good leadership and emotional demands, by defining good leadership as the best quartile instead of the best half and high emotional demands as those reporting “often” or more frequent.

## Results

Table 2 shows the characteristics of the study population, and the number of individuals entering antidepressant treatment in relation to these characteristics.

**Table 2:** Sample characteristics and caseness of antidepressant treatment in Denmark and Sweden

	Denmark		Sweden		Pooled	
	*n* (%)	Cases (%)	*n* (%)	Cases (%)	n (%)	Cases (%)
Total	6,096	266 (4.4)	3,411	141 (4.1)	9,507	407 (4.3)
Emotional demands and leadership						
High emotional demands						
Good leadership	1,098 (18.0)	58 (5.3)	874 (25.6)	51 (5.8)	1,972 (20.7)	109 (5.5)
Poor leadership	1,595 (26.2)	94 (5.6)	781 (22.9)	42 (5.4)	2,376 (25.0)	136 (5.7)
Low emotional demands						
Good leadership	1,805 (29.6)	67 (3.7)	920 (27.0)	22 (2.4)	2,725 (28.7)	89 (3.3)
Poor leadership	1,598 (26.2)	47 (2.9)	836 (24.5)	26 (3.1)	2,434 (25.6)	73 (3.0)
Sex						
Women	3,147 (51.6)	156 (5.0)	1,786 (52.4)	105 (5.9)	4,933 (51.9)	261 (5.3)
Men	2,949 (48.4)	110 (3.7)	1,625 (47.6)	36 (2.2)	4,574 (48.1)	146 (3.2)
Marital status						
Married/cohabiting	4,944 (81.1)	214 (4.3)	2,641 (77.4)	106 (4.0)	7,585 (79.8)	320 (4.2)
Not Married/cohabiting	1,152 (18.9)	52 (4.5)	770 (22.6)	35 (4.6)	1,922 (20.2)	87 (4.5)
Age (years)						
20–29	701 (11.5)	19 (2.7)	246 (7.2)	6 (2.4)	947 (10.0)	25 (2.6)
30–39	1,716 (28.2)	73 (4.3)	831 (24.4)	30 (3.6)	2,547 (26.8)	103 (4.0)
40–49	1,923 (31.6)	92 (4.8)	1,068 (31.3)	53 (5.0)	2,991 (31.5)	145 (4.8)
50–59	1,756 (28.8)	82 (4.7)	1,266 (37.1)	52 (4.1)	3,022 (31.8)	134 (4.4)
Education						
<10 years	509 (8.4)	26 (5.1)	473 (13.9)	13 (2.8)	982 (10.3)	39 (4.0)
10–11 years	671 (11.0)	49 (7.3)	798 (23.4)	33 (4.1)	1,469 (15.5)	82 (5.6)
12 years	826 (13.6)	36 (4.4)	758 (22.2)	29 (3.8)	1,584 (16.7)	65 (4.1)
13–14 years	2,426 (39.8)	89 (3.7)	486 (14.3)	29 (6.0)	2,912 (30.6)	118 (4.1)
at least 15 years	1,664 (27.3)	66 (4.0)	896 (26.3)	37 (4.1)	2,560 (26.9)	103 (4.0)
Wage						
Quartile 1 (lowest)	1,524 (25.0)	82 (5.4)	840 (24.6)	58 (6.9)	2,364 (24.9)	140 (5.9)
Quartile 2	1,524 (25.0)	75 (4.9)	851 (25.0)	36 (4.2)	2,375 (25.0)	111 (4.7)
Quartile 3	1,524 (25.0)	62 (4.1)	849 (24.9)	25 (2.9)	2,373 (25.0)	87 (5.7)
Quartile 4 (highest)	1,524 (25.0)	47 (3.1)	871 (25.5)	22 (2.5)	2,395 (25.2)	69 (2.9)
Full-time/part-time work						
Working part-time	1,433 (23.5)	67 (4.7)	590 (17.3)	33 (5.6)	2,023 (21.3)	100 (4.9)
Working full-time	4,663 (76.5)	199 (4.3)	2,821 (82.7)	108 (3.8)	7,484 (78.7)	307 (4.1)

Table 3 shows the main effects of emotional demands and quality of leadership on antidepressant treatment. Individuals with high emotional demands at work had an increased risk of entering antidepressant treatment with a pooled hazard ratio of 1.73 (95 % CI 1.41–2.13). Quality of leadership was not associated with entering antidepressant treatment.

**Table 3:** Incident antidepressant treatment in relation to main effects of emotional demands and quality of leadership

	HR^a^	95 % CI	*p* value for heterogeneity (*Q* test)
Emotional demands (high vs. low)	1.73	1.41–2.13	0.84
Leadership (good vs. poor)	0.94	0.78–1.15	0.69

*HR* hazard ratio, *95* *% CI* confidence interval

^a^Pooled estimate, adjusted for sex, age, marital status, education, wage, employment status (part-time vs. full-time)

Table 4 shows the joint effects of emotional demands and quality of leadership on antidepressant treatment. The hazard ratio for high emotional demands and poor leadership was 1.84 (95 % CI 1.32–2.57), whereas the hazard ratio for high emotional demands in combination with good leadership was 1.70 (95 % CI 1.25–2.31). The synergy index was 0.66 (95 % CI 0.34–1.28).

**Table 4:** Incident antidepressant-use in relation to joint distribution of emotional demands and quality of leadership

	HR^a^	95 % CI	Synergy index	95 % CI	*p* value for heterogeneity (*Q* test)
High emotional demands					
Good leadership	1.70	1.25–2.31	–	–	0.50
Poor leadership	1.84	1.32–2.57	–	–	0.26
Low emotional demands					
Good leadership	1.04	0.64–1.68	–	–	0.16
Poor leadership	1 (ref)	–	–	–	–
			0.66	0.34–1.28	0.51

*HR* Hazard ratio,* 95 % CI* confidence interval

^a^Pooled estimate, adjusted for sex, age, marital status, education, wage, employment status (part-time vs. full-time)

### Sensitivity analyses

The findings were similar for men and women, with pooled synergy indices of 0.74 and 0.83, respectively. The risk estimates for the effects of emotional demands was somewhat attenuated with adjustment for baseline mental health but remained statistically significantly elevated (HR = 1.49, 95 % CI 1.21–1.84, with *p* = 0.91 for heterogeneity). For quality of leadership, the hazard ratio after adjustment for baseline mental health was 1.17 (95 % CI 0.95–1.43) with *p* = 0.91 for heterogeneity.

Results were similar after adjustment for occupational group, with hazard ratios of 1.75 (95 % CI 1.41–2.17) with *p* = 0.54 for heterogeneity for emotional demands and 1.03 (95 % CI 0.85–1.25) with *p* = 0.51 for quality of leadership.

The country-specific estimates for the joint effects of emotional demands and quality of leadership on antidepressant treatment are shown in Table 5. The Danish risk estimate for high emotional demands and poor leadership was 2.11 (95 % CI 1.47–3.01), whereas the risk estimate for high emotional demands and good leadership was 1.86 (95 % CI 1.26–2.76). The synergy index was 0.63 (95 % CI 0.32–1.24). The Swedish risk estimates for high emotional demands were similar in the presence of poor and good quality of leadership, with HRs of 1.49 (95 % CI 0.92–2.41) and 1.47 (95 % CI 0.90–2.41), respectively. The Swedish synergy index was 1.83 (95 % CI 0.08–42.10), thus pointing in the opposite direction of the Danish index. The difference between the two synergy indices was not statistically significant.

**Table 5:** Country-specific estimates for incident antidepressant-use in relation to joint distribution of emotional demands and quality of leadership

	Danish estimates				Swedish estimates			
	HR^a^	95 % CI	Synergy index	95 % CI	HR^a^	95 % CI	Synergy index	95 % CI
High emotional demands								
Good leadership	1.86	1.26–2.76	–	–	1.47	0.90–2.41	–	–
Poor leadership	2.11	1.47–3.01	–	–	1.49	0.92–2.41	–	–
Low emotional demands								
Good leadership	1.27	0.87–1.84	–	–	0.77	0.43–1.37	–	–
Poor leadership	1 (ref)	–	–	–	1 (ref)	–	–	–
			0.63	0.32–1.24			1.83	0.08–42.10

*HR* hazard ratio, *95* *% CI* confidence interval

^a^Adjusted for sex, age, marital status, education, wage, employment status (part-time vs. full-time)

When changing the cut-off points and defining good leadership as the best quartile and high emotional demands to those responding “often” or always, some indication of buffering was seen in the Danish data: the HR for high emotional demands with poor leadership was 1.58 (95 % CI 1.10–2.25), whereas the HR with good leadership was 1.03 (95 % CI 0.72–1.46). However, the Swedish results pointed in the opposite direction, with a HR for high emotional demands and poor leadership of 1.17 (95 % CI 0.71–1.93) and for high emotional demands and good leadership of 1.41 (95 % CI 0.86–2.31). It should be noted, though, that the numbers of respondents now classified as having high emotional demands and good leadership were small; there were 139 (6 cases) and 95 respondents (7 cases) in this category in DWECS and SLOSH, respectively.

## Discussion

This study examined whether good leadership buffers the effects of emotional demands on common mental disorders, as indicated by antidepressant treatment. The results, however, indicate that any buffering by good leadership is modest; the risk of entering antidepressant treatment was increased for employees with high emotional demands, whether leadership was poor 1.84 (95 % CI 1.32–2.57) or good 1.70 (95 % CI 1.25–2.31). Also, the synergy index was not statistically significantly different from 1, although this could be due to lacking statistical power. The findings were unchanged when defining only the best quartile as good leadership, so the patterns are unlikely to be explained by the dichotomization of leadership quality. The country-specific estimates for Denmark and Sweden showed somewhat differing patterns with some indication toward buffering in the Danish data; antidepressant treatment was, however, still substantially increased for Danish employees with high emotional demands and good leadership, indicating that any preventive effects of leadership may be modest.

To the best of our knowledge, this is the first study to examine the buffering effect of leadership quality on the association between emotional demands at work and antidepressants. The lack of buffering contrasts the conservation of resources theory [19], which states that high availability of resources reduces the risk that expenditure of resources results in adverse health outcomes. The measure we used for leadership quality was, however, partly based on the availability of similar items in the two studies. Hence it was not a validated scale, and may not have captured the most important aspects of leadership quality satisfactorily, possibly explaining the lack of main and buffering effects of this construct in the present analyses. Another limitation of our study is that we measured emotional demands using a single item, rather than the full scale––again this operationalization reflected data availability. Furthermore, the general population study sample we applied may not have provided sufficient exposure contrast on leadership quality to demonstrate its full buffering potential, given the distribution of this construct in the sample, with most respondents clustered around average-to-good levels. The issues concerning the exposure contrast of both emotional demands and leadership were not improved by their dichotomizations, which were unfortunately required to examine buffering as departure from additivity. It seems that further studies examining occupation- and/or workplace-specific samples may be needed on this issue.

In relation to the measurement of leadership, the applied scale largely measured supportive leadership, and indeed two of the items in the Danish data were previously used in a scale on supervisor social support. Although we had reason to expect that social support could buffer the consequences of stressors [22], it is possible that social support from colleagues may be more important than social support from supervisors, in the context of emotional demands. Whilst we could not examine the relevance of colleague social support in the present study, due to data availability, this issue certainly warrant further scrutiny.

Although we did not find substantial buffering of the association between emotional demands and antidepressants by quality of leadership, this should not be interpreted as a negation of the importance of ensuring good leadership at work; previous research indicates that managers who provide information, clarity, support, and inspiration are favourable to employee health [43, 44]. Better mental health has also been associated with transformational leadership, i.e. leadership characterised by idealised influence, inspirational motivation, intellectual stimulation, and individualised consideration [45]. Furthermore, employee psychological well-being has been found increased with leaders who provide feedback, support, communication, commitment to quality, fairness, integrity/respect, participation and empowerment, and value diversity [20]. Reduced employee psychological well-being, conversely, has been associated with leadership that is experienced as autocratic, malevolent, and self-centred [46]. Additionally, longitudinal intervention-based evidence suggests that strengthening the leaders’ abilities to intervene in conflict-ridden interpersonal situations leads to improved employee psychological well-being [47]. Some, but not all, of these qualities were measured in the present study, and the applied leadership measure possibly explains the lacking main effect of leadership on antidepressants.

Alternatively, the lack of buffering may be explained by opposite effects of leadership on mental health and treatment-seeking in individuals with emotionally demanding work. If good leaders are associated not only with better employee mental health but also more supportive of treatment seeking, these effects may balance each other out, when examining antidepressant treatment.

It is, however, also possible that predictors of clinically significant common mental disorder differ from those of self-reported mental well-being or psychological distress––the outcomes most often previously associated with leadership quality. Consequently, it seems that more work is needed to identify health promoting leadership aspects and their effects [43].

Emotional demands at work have previously been associated not only with antidepressant treatment [15, 16] but also other mental health indicators, including hospitalization with depression [14] and depressive symptoms [12, 13]. It is unclear, though, if there is a causal effect of emotionally demanding work on mental health. The association may be confounded by poor baseline mental health possibly increasing the risks of experiencing high emotional demands at work and developing common mental disorder. The results in this article, however, suggest that this may not be a primary source of bias. First, emotional demands were still associated with antidepressant treatment after adjustment for baseline mental health. Second, if poor baseline mental health influences how employees experience their work, this would likely influence both the experience of high emotional demands and poor leadership quality. Hence, if baseline mental health were the primary force driving the associations between work environment and common mental disorders, we would not expect an increased risk of antidepressant treatment in the group experiencing high emotional demands but good quality of leadership. Consequently, the observed pattern with increased antidepressant treatment in employees with high emotional demands regardless of leadership quality, suggests that baseline mental health differences are not the primary factor driving the association. This pattern, would, however, also be consistent with a selection of individuals particularly vulnerable to common mental health problems into emotionally demanding occupations, as indicated previously for Danish eldercare workers [48]. Such selection could, however, not be partialled out given the available data. Hence, the causality of the association between emotional demands and mental health remains uncertain.

Another potential confounder when using antidepressants as an indicator for common mental disorders is treatment-seeking behaviour [49]. Emotional demands are high in human service occupations (e.g., nursing, teaching) [15]. If these employees are also more likely to seek medical help when suffering from common mental disorder, this would bias the observed associations upwards. The sensitivity analysis adjusting for occupational group did, however, not change results––suggesting that occupation is not a major confounder for the results of this article. It should be noted, though, that the ISCO-88 major grouping is somewhat coarse and may not have been sufficiently nuanced to fully account for any occupational differences.

This study used national data on purchases of antidepressant medications, as an indicator of clinically significant common mental disorder. This allowed a 2.6-year day-to-day follow-up of all participants from the two studies and avoided selective participation at follow-up. The outcome of antidepressants treatment as an indicator for common mental disorder should, however, be interpreted cautiously. Antidepressants are used for various disorders, not only including depressive, anxiety, and sleep disorders, but also incontinence, bulimia, headache, and psychotic disorders [50, 51]. As most antidepressants are, nevertheless, prescribed for common mental disorders [51–53], antidepressants seem a valid measure of common mental disorder in general, but specific disorders cannot be disentangled. Unfortunately, the treatment indication was unavailable in the present study. Hence, the association between emotional demands and common mental disorder should be examined further using more specific outcome measures that might point to the exact nature of any mental health consequences.

To conclude, we found that high emotional demands at work predicted entering antidepressant treatment and that good leadership did not convincingly prevent this effect. Further research is needed to identify possible buffers of effects of emotional demands at work on employee mental health.
